# Can cognitive enhancers reduce the risk of falls in older people with Mild Cognitive Impairment? A protocol for a randomised controlled double blind trial

**DOI:** 10.1186/1471-2377-9-42

**Published:** 2009-08-12

**Authors:** Manuel Montero-Odasso, Jennie L Wells, Michael J Borrie, Mark Speechley

**Affiliations:** 1 Department of Medicine, Division of Geriatric Medicine, Parkwood Hospital, University of Western Ontario, London, ON, Canada; 2 Department of Epidemiology and Biostatistics, University of Western Ontario, London, ON, Canada; 3 Lawson Health Research Institute, London, ON, Canada

## Abstract

**Background:**

Older adults with cognitive problems have a higher risk of falls, at least twice that of cognitively normal older adults. The consequences of falls in this population are very serious: fallers with cognitive problems suffer more injuries due to falls and are approximately five times more likely to be admitted to institutional care. Although the mechanisms of increased fall risk in cognitively impaired people are not completely understood, it is known that impaired cognitive abilities can reduce attentional resource allocation while walking. Since cognitive enhancers, such as cholinesterase inhibitors, improve attention and executive function, we hypothesise that cognitive enhancers may reduce fall risk in elderly people in the early stages of cognitive decline by improving their gait and balance performance due to an enhancement in attention and executive function.

**Method/Design:**

Double blinded randomized controlled trial with 6 months follow-up in 140 older individuals with Mild Cognitive Impairment (MCI). Participants will be randomized to the intervention group, receiving donepezil, and to the control group, receiving placebo. A block randomization by four and stratification based on fall history will be performed. Primary outcomes are improvements in gait velocity and reduction in gait variability. Secondary outcomes are changes in the balance confidence, balance sway, attention, executive function, and number of falls.

**Discussion:**

By characterizing and understanding the effects of cognitive enhancers on fall risk in older adults with cognitive impairments, we will be able to pave the way for a new approach to fall prevention in this population. This RCT study will provide, for the first time, information regarding the effect of a medication designed to augment cognitive functioning have on the risk of falls in older adults with Mild Cognitive Impairment. We expect a significant reduction in the risk of falls in this vulnerable population as a function of the reduced gait variability achieved by treatment with cognitive enhancers. This study may contribute to a new approach to prevent and treat fall risk in seniors in early stages of dementia.

**Trial Registration:**

The protocol for this study is registered with the Clinical Trials Registry, identifier number: NCT00934531 http://www.clinicaltrials.gov

## Background

### Cognitive Decline and Falls: A well-known couple

An important goal of geriatric medicine is to reduce the gap between life expectancy and disability-free life expectancy. A substantial portion of this gap is caused by two major geriatric syndromes: cognitive impairment and mobility limitation, which ultimately manifest as dementia and falls, respectively. Interestingly, these manifestations often coexist in elderly people: falling is a common geriatric syndrome affecting about a third of older adults each year, and dementia has a prevalence of 8% of Canadians aged 65 and 35% in people over age 85 [1-3]. This interrelationship has been attributed to specific brain networks selectively affected by diseases that accompany, but are not necessarily caused by, ageing [4].

Older adults with cognitive problems have a higher risk of falls, with annual incidence of around 60–80%; at least twice that of cognitively normal older adults [5]. The consequences of falls in this population are very serious; fallers with cognitive problems are approximately five times more likely to be admitted to institutional care than people with cognitive issues who do not fall [6]. They are also at high risk of major fall-related injuries such as fractures and head injuries leading to increased mortality. Falls are a major cause of disability and dependence in older people, and more so for those with cognitive problems. In addition to indirect costs and caregiver burden, the direct costs of emergency, acute, rehabilitation and long-term care are substantial and increasingly unsustainable for the health care system. Although the mechanisms of increased fall risk in cognitively impaired people are not completely understood, it is known that impaired cognitive abilities can reduce attentional resource allocation while walking [7]. As well, since executive function is an important cognitive resource for normal walking, impairments in this domain are also associated with both dementia and risk of falls [8].

One approach to mitigating fall risk in people with memory problems is to target them in the early stages of cognitive decline. Mild Cognitive Impairment (MCI) is a recognized clinical entity that is a transitional state between benign age-related cognitive change and early dementia. Specific diagnostic criteria have been developed and validated [9-13] to diagnose MCI, with the prevalence of the diagnosis being estimated at 19% among older adults, increasing to 29% in those over age 85 [14]. People with MCI have been found to have a 10 to 15 times higher risk of developing Alzheimer's disease (AD), as well as a higher risk of falling compared with age-matched controls [15,16].

### The role of cognition on gait: The dual-task paradigm and gait variability

Although walking has long been considered primarily as an automatic motor task, emerging evidence suggests that this view is overly simplistic [17]. Cognitive function may play a key role even in the regulation of routine walking, particularly in older adults. Attention is a necessary cognitive resource for maintaining normal walking and there is evidence that cognitive and attentional deficits are independently associated with postural instability, impairment in performing daily living activities, and future falls [8]. The role of cognition in walking is even more marked in people with cognitive dysfunction, whose gait performance is affected by any extra cognitive load. Since Lundin-Olsson and colleagues' seminal "stops walking while talking" study [18] demonstrated that the inability to maintain a conversation while walking is a marker for future falls in older adults, observing people walking while they perform a secondary task ("dual-task paradigm") has become the accepted way to assess the interaction between cognition, gait, and risk of falling. Previous research on the effect of dual-tasking on gait performance showed specific associations between slowing gait and executive dysfunction and attention deficits [15,17,19-25]. In a previous study, we assessed 60 elderly individuals with MCI and found that impairments in several cognitive domains (attention, executive function, and working memory) are associated with both a slow usual gait velocity and slower gait velocity under dual-task conditions, demonstrating that these specific cognitive domains are crucial for maintaining normal gait performance [26].

A sensitive measure of gait performance is gait variability, defined as the stride-to-stride variation in time [27]. This measure quantifies the automaticity of gait, with greater variability indicating less rhythmicity and a more unstable gait pattern. Evaluating gait variability is an accurate methodology to identify subtle changes in walking due to pathological conditions or disease. For instance, cognitively normal older adults have low gait variability; however, high gait variability has been described in Parkinson's disease, Alzheimer disease, and has been associated with high risk of future falls and mobility decline [28]. Additionally, previous studies have demonstrated that gait variability may serve as a clinically relevant parameter in the evaluation of mobility, and may be a responsive measure for different interventions in fall prevention [29].

### There is a lack of effective strategies for preventing falls in older people with memory problems

Previous trials in cognitively normal older adults have demonstrated that both multifactorial (e.g. review of medications, strength and balance training, visual and hearing corrections, and environmental modifications) and some single interventions (resistance and balance exercises) are effective in preventing falls [30]. By contrast, most of the studies targeting falls in people with cognitive problems have been unable to prevent falls [31-33].

A recent systematic review and meta-analysis [31] concluded that the benefit of these single and multifactorial interventions does not translate from cognitively normal older adults to those with dementia. It is possible that different underlying mechanisms are at work in those with dementia; thus, a different approach may be necessary to target fall risk in this population. Although much is known about the multifactorial nature of falls in cognitively normal individuals, knowledge about the nature of falls in those with cognitive problems is limited and as a consequence, the number of falls and fall related injuries in this population continues to increase [33].

### Pharmacological Treatment for Cognitive Problems

Cholinesterase inhibitors (ChEI), although modest in effect, are the most useful pharmacological treatment available for Alzheimer's Disease and vascular dementia [2,34]. Although not curative, their effects include memory stabilization and delays in functional decline and nursing home placement [2].

**Figure 1:**
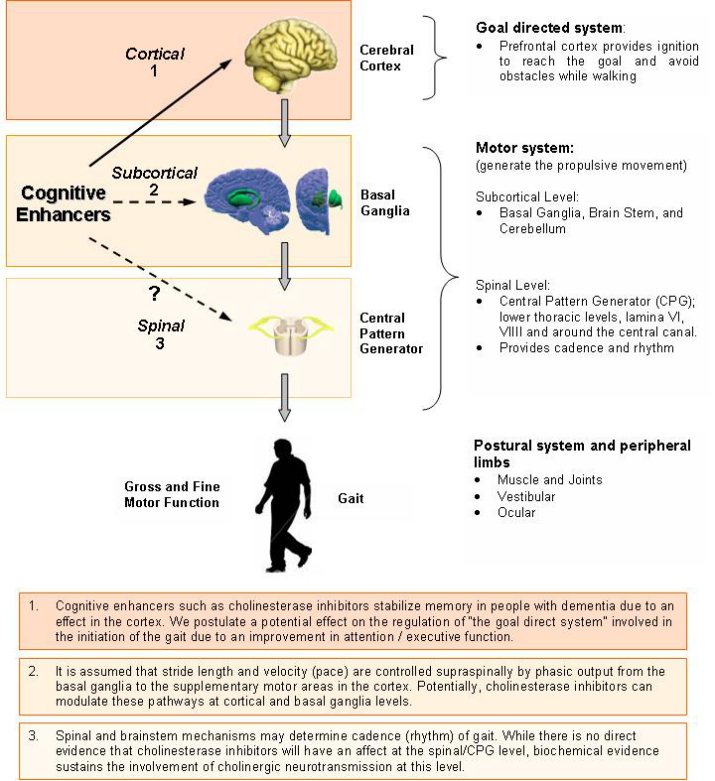
**Regulation and Neural Control of Gait**.

The molecular mechanism of action of ChEI is through increased cortical and hippocampal acetylcholine, an important neurotransmitter for memory regulation and neural plasticity. However, the mechanism of the clinical improvements in delaying functional decline is not well understood. One possible explanation may relate to not only the cognitive action of the drug, but also to subtle improvements in the motor function of these patients. Whether by direct effect or mediated through cognition, motor function improvement would consequently serve to stabilize mobility and delay functional decline. In Figure 1, we propose possible levels of action of ChEI on motor function and gait.

Cholinesterase inhibitors have also been tried in individuals with MCI, with the goal of delaying the progression to dementia. A recent RCT demonstrated that cognitive enhancers might improve cognition in this population; however, the effect was weak and had questionable clinical significance [12]. Currently, there is no indication to use cognitive enhancers to treat people with MCI with the goal of delaying or preventing further functional or mobility decline.

Recently, it has been suggested that ChEI may improve gait performance [35] through an improvement in attentional resource allocation due to the fact that ChEI are known to improve attention and executive function [34]. Besides this effect on mobility through cognition, cholinergic neurotransmission has a potential effect on mobility and gait regulation not mediated through cognitive improvement since cholinergic neurons in the striatum are involved in movement and motor functions. For instance, dysfunction of cholinergic neurons in the striatum is known to occur in movement disorders (such as Huntington's chorea), and an imbalance between dopaminergic and cholinergic transmission is found in Parkinson's disease and related drug-induced dyskinesias [36]. These related findings lend both clinical and mechanistic plausibility to the hypothesis that cognitive enhancers may also have a potential effect on motor function and gait.

### Effect of cognitive enhancers on motor function

Despite the well-recognized effect of cognitive enhancers on cognitive status, there is a shortage of studies investigating their effect on motor functioning, especially on walking, which will most likely have an impact on fall risk in this population. A recent pilot study has shown that fine motor skills as evaluated by hand movements improved with cognitive enhancer treatment [37]. This was an open label study in 12 patients with Alzheimer's Disease (AD) using the ChEI, donepezil, at 5 mg/day for 4 weeks then increased to 10 mg/day for 8 weeks. The investigators noted a trend toward improved (i.e. faster) finger tapping scores following donepezil treatment. Similarly, a case series showed that individuals with dementia taking galantamine (a cholinesterase inhibitor similar to donepezil) for 24 weeks had less decline in gait performance compared with age matched controls [35]. Nevertheless, there have been no studies to date evaluating the effect of ChEI on fall risk, balance confidence, and gait variability in elderly people with Mild Cognitive Impairment.

### Pilot data and Rationale

**Figure 2:**
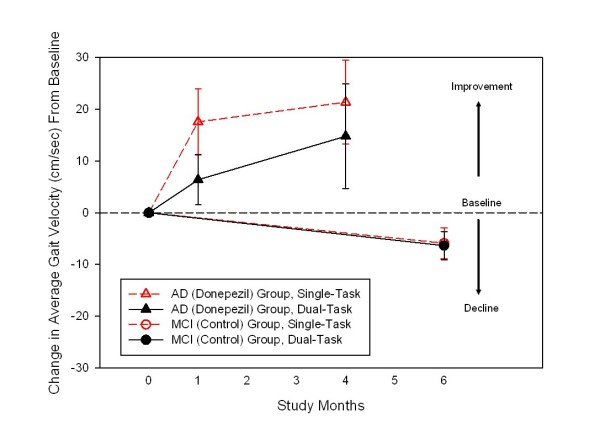
**Changes in Gait Velocity in people with early Alzheimer's disease (AD) taking donepezil (intervention group) and Mild Cognitive Impairment (MCI; control group)**.

As a proof of principle for this proposed RCT, we assessed the effect of donepezil, over four months of treatment, on gait using an electronic walkway (GAITRite System) in six individuals with AD [38]. An increase in gait velocity and a reduction in gait variability were seen following cognitive enhancer treatment. In brief, participants with AD taking donepezil increased their mean gait velocity (G-vel) after one month (from 89.7 ± 11 to 106.9 ± 22, *p *= .045) under a single-tasking condition. These increases were sustained and improved after four months, suggesting a dose-response pattern as shown in Figure 2. Gait variability (G-var), assessed as coefficient of variation of stride time, decreased during follow-up (single-task from 22.3 to 11.30, *p *= .04). Since it is known that gait performance declines over time in older people, we compared these results with observational data from eight comparison individuals with MCI and found that the MCI group experienced a reduction of gait velocity over time. (Figure 2)

Although the small sample size and the lack of randomization are limitations of this open label study, these preliminary findings provide preliminary data and rationale for pursuing the current clinical trial. We will assess whether donepezil treatment will improve gait performance under single and dual-task conditions in older individuals with MCI by comparing them with MCI controls receiving placebo. Additionally, we will test the effect of the intervention on two well-established clinical markers of fall risk such as balance sway and the balance confidence scale (ABC) [39], and also on the actual number of falls. All assessments will be completed over a 6-month follow-up period.

Currently, there is no clinical indication for prescribing cognitive enhancers in people with MCI, so there is genuine equipoise about the effect of cognitive enhancers on gait, balance, and risk of falls in this population.

## Methods/Design

### Design

**Figure 3:**
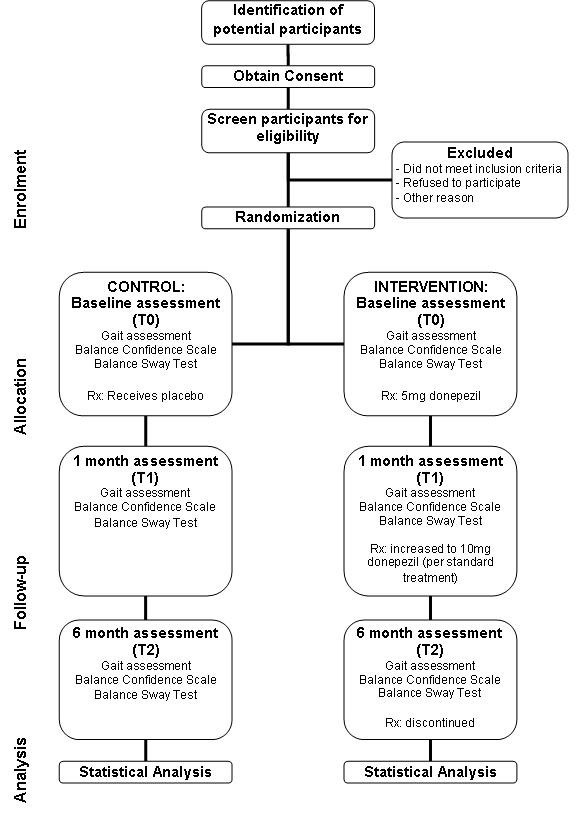
**Trial Design**. Adapted from the CONSORT diagram [61,62].

Double blinded randomized controlled trial with 6 months follow-up in 140 older individuals with MCI. Participants will be randomized, in blocks of four, to the control and the intervention groups (Figure 3).

### Participant Selection

Our pilot study referenced above has given us preliminary research experience with this patient population. Participants will be recruited from the Aging Brain and Memory Clinic at Parkwood Hospital. The clinical investigators will enrol an average of five (5) participants per week, thereby recruiting the 140 participants over a period of 7 months. In order to maximize enrolment possibilities, the recruitment period will be extended to 10 months if the 140 participants are not reached at month 7.

#### Inclusion criteria

• patients who have MCI (diagnosed using criteria suggested by Winblad et al [13])

• Aged 65 years and older

• Able to walk independently 10 meters without any gait aid (for example: walker, cane)

• Able to travel to the clinic for the assessments

#### Exclusion criteria

• Unable to understand English

• History of psychiatric illness within the last two years, including depression

• Parkinsonism or any neurological disorder with residual motor deficit (e.g. stroke, epilepsy)

• Musculoskeletal disorder detected by clinical examination which affects gait performance

• Active osteoarthritis affecting the lower limbs (America College of Rheumatology criteria [40-42])

• Chronic Bradycardia (baseline heart rate below 60 beats/minute)

• Use of psychotropic medication, which can affect motor performance (e.g. neuroleptics and benzodiazepines)

• Depression (score above 8/15 on the Geriatric Depression Scale – GDS [43,44]) since depression has been shown to reduce gait performance.

In order to increase generalizability of the results, the inclusion/exclusion criteria have been restricted to factors that can compromise study adherence and gait evaluation. The exclusions are to reduce the statistical noise introduced by specific diseases or disabilities that have strong known effects on gait. Exclusions will be based on a formal medical examination. The diagnosis of MCI is based on clinical criteria [15,45], which includes the presence of subjective memory complaints from the patient and family, objective memory impairment, preserved general intellectual function assessed clinically, absence of significant functional impairment, and absence of clinical dementia as assessed by a skilled geriatrician with extensive experience in a memory clinic setting. Objective memory impairment is operationalized, following the criteria set out by Petersen and colleagues [11]; demonstrated by memory impairment judged as beyond the normal range for age and education by a skilled geriatrician. Additionally, global cognition will be assessed using the Mini Mental State Examination (MMSE; scored 0–30), and the Montreal Cognitive Assessment (MoCA; scored 0–30, with higher scores indicating better performance). The MoCA test is a validated tool used to assess global cognition and was originally created to assist in the diagnosis of MCI [46]. In brief, when considering MMSE and MoCA performance in the same individual, a pattern of low MoCA score (<26) with normal MMSE score (>26) is associated with the diagnosis of MCI [46].

The Health Sciences Research Ethics Board at The University of Western Ontario approved the research protocol – Research Protocol Number 16086. This RCT has been registered in clinical trials.gov (identifier number: NCT00934531)

### Measurements and Procedures

**Figure 4:**
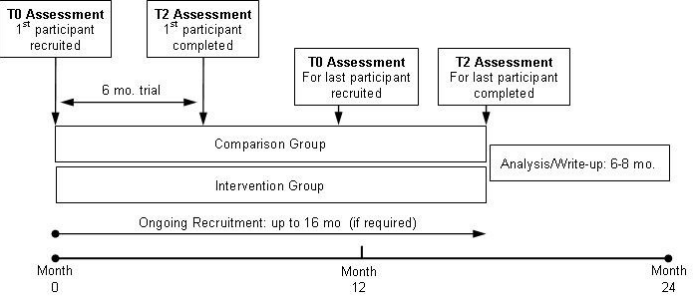
**Schematic timeline of the proposed RCT**.

All participants will undergo a baseline, one month, and a final six-month assessment. Baseline gait assessment will occur within one week prior to starting donepezil (T0), with a second assessment occurring after 4 weeks of treatment at 5 mg of medication (T1). The third and final assessment will occur after participants have been on the full dose of donepezil, 10 mg, for a period of 5 months (T2), yielding a total period of follow-up of 6 months from baseline. Assessments at each of these time-points will include gait analysis, balance sway, and balance confidence as well as cognitive measurement of attention and executive function. Falls will be retrieved by phone interview as has been done in previous studies [47,48]. A timeline for the proposed study can be found in Figure 4.

#### Hypothesis and Objectives

• **Hypothesis 1: **Cognitive enhancers reduce fall risk in elderly people with MCI by improving their gait performance.

• **Hypothesis 2: **Cognitive enhancers improve balance and balance confidence in elderly people with MCI.

• **Mechanistic Hypothesis: **The improvement on gait and balance measures is due to an enhancement in attention and executive function.

• **Objective 1: **To assess the effect of donepezil on gait velocity and gait variability in elderly people with MCI using an electronic walkway (Gait Rite System, CIR Systems Inc.).

• **Objective 2: **To evaluate the effect of donepezil on balance and in balance confidence in elderly people with MCI using a balance platform (Bertec Inc.) and the Activities-Specific Balance Confidence Scale (ABC).

• **Objective 3: **To test if these potential improvements on gait and balance are mediated through an enhancement in attention and executive function.

### Outcomes measures

#### Primary outcomes

1. Improvements in gait velocity (cm/second) at month six

2. Reduction in gait variability assessed as standard deviation (SD) and coefficient of variation (CoV) at month 6.

#### Secondary outcomes

Between-group improvement in:

1. Balance confidence as evaluated by the Activities-Specific Balance Confidence Scale (ABC)

2. Balance sway, maximum saggital displacement, using a force platform (Bertec Inc.)

3. Attention measured with the Digit Span Test

4. Executive function using the Trail Making Test, parts A and B

5. Reduction of number of falls: The total number of falls by month 6 (T2) and proportion of participants who fall are also secondary outcome measures to be evaluated.

### Randomisation

Once informed consent has been obtained and the baseline assessment has been completed, participants will be allocated to one of the two arms at a ratio of 1:1. Stratified randomization within strata based on fall history (0 falls, 1+ fall in past 12 months) will be performed. A block randomization by four will be applied to ensure a cumulative balance of participants between arms. The randomization sequence of the participants will be generated by a computer program and allocation to groups will be done with sequentially numbered opaque sealed envelopes. A professional bio-statistician not involved in recruitment and assessment will perform the randomization

### Follow-up

As shown in the timeline (Figure 4), participants will be followed for 6 months after their baseline measurements. Improvements in the primary outcomes of interest are expected after 4 months of treatment based on our pilot study and on a previous report of the potential effect of cognitive enhancers on gait and mobility. However, a six-month follow-up will increase the probability of finding a significant reduction of the number of falls and the adverse events because of the intervention. The three assessments will be conducted by trained research assistants and all the questionnaires will be checked for missing data and completed with the participants under their supervision.

### Evaluations

Inclusion/exclusion criteria will be verified through a standardized neurological and musculoskeletal examination by the principal investigator.

#### Instrumentation

Gait performance will be assessed using an electronic walkway system (GAITRite^®^) under single and dual-task conditions. The GAITRite^® ^system includes a portable electronic walkway mat (600 cm in length and 64 cm in width) for the automated measurement of spatiotemporal gait parameters. As participants walk along the mat, imbedded sensors are activated by the pressure of their feet and deactivated when the pressure is released. A computer processes the footsteps, providing data for both spatial and temporal parameters. Gait parameters are recorded using only the footprint of the participants, thereby eliminating the need for external sensors attached to the body or lower limbs that may interfere with gait performance or may confuse an older individual with a cognitive impairment. The mat is located in a well-lit, 10-meter long hallway at the Division of Geriatric Medicine at Parkwood Hospital. Start and end points will be marked on the floor with tape one meter from either end of the mat to avoid recording acceleration and deceleration phases.

#### Gait Assessments

Participants will be given standardized instructions and a visual demonstration of the different gait task. Participants will then be asked to perform three single-task trials and three dual-task trials. The single task trials consist of walking the length of the mat at a self-selected pace. For the dual-task trials, participants will be asked to walk while counting aloud backward from one hundred by ones. This dual-task condition is selected based on previous research which demonstrated that counting backwards requires both working memory and attention [49]. Participants will be instructed to pay attention to both gait and the cognitive task; if a participant stops either task during the trial they will be prompted to continue. Allowing both gait and cognitive task to vary has previously been shown to provide a better representation of what happens naturally [8,50].

#### Balance Confidence Scale

Balance confidence will be evaluated using the Activities-Specific Balance Confidence Scale (ABC) [39]. Respondents self-rate their confidence about their balance while performing a series of daily tasks on this 16-item questionnaire. The described tasks range in difficulty from those of basic daily living (e.g. walking around the house, going up and down stairs), to more difficult tasks generally performed in the community (e.g. walking in crowded areas like shopping centres, using escalators). Respondents are asked to rate their confidence on a scale from 0% (no confidence) to 100% (complete confidence) based on the following cue question: "How confident are you that you will not lose your balance or become unsteady when you [list of items follows]." The scale's wide range of item difficulty makes it well suited to assessing balance as a construct in populations with varying levels of functioning, including high-functioning community living seniors. This scale has been validated in previous studies as a marker of risk of falling [39,51].

#### Balance Sway Test

Balance will be quantified as body sway using a Digital Balance Platform available in our service (Bertec Inc.). Displacements of the body in frontal and saggital direction will be recorded in millimetres. Peak-to-peak amplitude (maximum displacement of center of pressure, CoP) in the saggital and frontal direction will be calculated. Sway area will be calculated by multiplying the maximal frontal diameter with maximal saggital diameter following the method validated by Lord and colleagues [52]. Testing will be performed with subjects standing directly on the balance plate and standing on a foam rubber mat placed on the balance plate, with both measures being repeated with the participants having their eyes open and closed.

#### Falls

A fall is defined as 'an unintentionally coming to rest on the ground, floor, or other lower level and not due to a seizure or an acute stroke [63]. Recurrent falls are defined as two or more events in a six-month period during the trial. An absolute reduction of 10 points in the rate of falls is expected in intervention group after six months of follow-up (20% in the control to 10% in the intervention). Because falls tend to be forgotten if no injuries are involved, a fall calendar enabling prospective assessment will be implemented [64]. At the baseline session, participants will receive a fall calendar and instructed in its use (i.e. they will be asked to record every day whether they have had a fall). Each monthly calendar page detaches along a perforation and becomes a postage-paid postcard addressed to the study office. Calendar pages each have a unique study ID number and no identifying information. If a calendar is not received by two weeks into the following month, or is missing or incomplete the research assistant will contact the participant by phone and together they will complete the page. Participants who indicate one or more fall will be contacted by study staff and interviewed about the circumstances and outcomes of the falls. These methods have been used locally in previous research [47,48].

#### Data acquisition of the quantitative gait variables

GAITRite software Version 3.8 will be used to process the footstep data using an algorithm for light and short footsteps as older individuals with cognitive problems may be more likely to slow down or hesitate while dual-tasking. Participants will be instructed to wear comfortable clothes and shoes. Gait variability will be analyzed in terms of stride time variability, and reported as coefficient of variation [27]. Validity and reliability of the GAITRite system has been established in older adults with memory problems by ourselves [53] and other research groups [54-56].

### Sample size determination

Sample size determination is based on our primary outcomes and using the values from the pilot data described above. We expect a 10% improvement in gait velocity (G-vel), measured in cm/s (effect size of 0.10 m/s, from 1.0 m/s to 1.10 m/s), and a reduction in gait variability (G-var) (stride time variability) of 10% as assessed by decreases in standard deviation and coefficient of variation. These expected changes are based on our preliminary data which showed that mean G-vel increased by 15% after one month of treatment and these changes were sustained and improved in the 4 months follow up [38]. In this study, we found that the mean stride time at usual gait was 1190 ± 266 milliseconds (ms), after one month of treatment was 1053 ± 178 ms, and after 4 month was 999 ± 112 ms. The absolute reduction of the mean group CoV was 11 points (from 22.4% to 11.3%). Therefore, it is reasonable to expect similar improvements in our proposed study with a larger sample. Thus, in order to detect a group reduction of variability of 10% with a type II error of 0.20, 69 participants in each arm will be needed for the final analysis; assuming a 5 percent drop-out rate (which was seen after 6 months in two previous studies testing medications on MCI), 75 per arm participants will need to be recruited. This sample will also allow the detection of a 10% difference in the mean scores of the ABC, which is considered clinically significant. While we will not have sufficient power for a definitive analysis of changes in fall rate or risk, we will be able to compare changes in the mean values of the primary outcomes with between-group differences in fall rate. This will add mechanistic evidence that changes in fall rate are due to changes in gait parameters, and will provide a point estimate needed for the sample size calculations for a future trial powered to detect a reduction in falls as a primary endpoint.

### Statistical Analysis

Analyses will be performed according to an intention-to-treat principle. Baseline characteristics and gait parameters will be descriptively summarized using either means and standard deviations or frequencies and percentages, as appropriate. Comparisons of the changes in the mean balance confidence score will be assessed using *t*-test. The coefficient of variation [CoV = (SD/mean)*100] will be used to quantify gait variability. We will use linear regression to perform between-group comparisons using three data points (baseline, one month, six months) with baseline – 1 mo. corresponding to before treatment and 1 mo. – 6 mo. corresponding to treatment with donepezil. Linear regression models, both unadjusted and adjusted for age, sex, and history of falls, will be used for comparison of individual variables. Using the baseline value of an endpoint as a covariate is more powerful statistically than comparing the differences from baseline. The statistical significance of the results will be determined by Hochberg's variation of the Bonferroni procedure for multiple testing [65]. Two-sided *p *< 0.05 will be considered statistically significant. All calculations will be made using SPSS software package version 16.0 (SPSS Inc., Chicago, IL).

## Anticipated problems and contingency plans

### Recording falls

Since falls tend to be forgotten or underreported, a strict definition of falls and a fall calendar will be implemented as "unintentionally coming to rest on the ground or other lower level." Participants will be asked to record any falls on the calendar every day and mail the completed calendar to the study office at the end of each month. Research assistants will verify the accuracy of all positive responses by phone interview, and will contact participants if calendars are more than two weeks late being mailed in. This strategy has been proven to be very effective for fall recording by others and us [47,57,58].

### Tolerance to the medication

Donepezil is judged to be fairly well tolerated in older people at the 10 mg/day standard dose regimen. We selected Donepezil for our study since, according to the last Cochrane Review on cholinesterase inhibitors [59], fewer patients suffer adverse events on Donepezil when compared with other cognitive enhancers such as Rivastigmine. The most common reported intolerance is due to gastrointestinal problems such as abdominal pain, anorexia, nausea, vomiting, diarrhoea, and rarely dizziness, headache, and insomnia, which were significantly more frequent in the ChEI than in placebo groups in the referenced meta-analysis. Participants who would develop gastrointestinal intolerance with Donepezil at 10 mg/day will have the dose reduced to 5 mg/day and switched to a bedtime regimen as per current clinical practice. Participants who have persistent intolerance, and anyone who experiences a major adverse reaction, will be withdrawn from the study and the events reported to the Research Ethics Board and to Health Canada.

### Effect of Donepezil on balance

The last Cochrane review and meta-analysis [59,60] on adverse events associated with cholinesterase inhibitors showed that adverse events such as gait disturbances, falls or balance problems are not significantly more frequent in the ChEI groups than in placebo. These findings were pooled evidence from six or more studies in the referenced meta-analysis. In our ongoing pilot project involving 16 participants with early dementia we have not found any participants who experienced adverse events related to balance, bradycardia, or hemodynamic changes. As per clinical practice, participants having bradycardia before randomization (baseline heart rate below 60 beats/minute) will be excluded of the study. Therefore, based on published evidence and our accumulating direct experience, we do not expect a significant negative effect of ChEI in balance and/or hemodynamic changes in our population.

## Discussion

Elderly adults with cognitive problems are one of the most vulnerable sectors of our society with clear mental, social, and physical disadvantages. They are more likely to experience falls, and experience further mobility decline due to having fallen; therefore is an urgent need to identify evidence based interventions for reducing the risk of falls and related injuries in people with cognitive impairment. To date, no adequately- powered studies have investigated the effect of cognitive enhancers to reduce falls in people with MCI. As falls are one of the most common reasons for both hospital admissions and nursing home placements in older adults with cognitive difficulties, they contribute significantly to the overall health care burden for Canada.

This study is distinctive due to the fact the intervention will target cognition with the outcome to improve mobility, balance, gait performance, and risk of falls. If we can demonstrate the effect of cognitive enhancers on fall risk and gait performance, these results will reveal a previously unrecognized benefit of this pharmacological therapy, which could in turn serve to decrease this burden. We are a unique position to conduct this project based on our previous research program expertise with gait assessment as well as our record of research involving elderly people with MCI

By characterizing and understanding the effects of cognitive enhancers on fall risk in older adults with cognitive impairments, we will be able to pave the way for a new approach to fall prevention in this population. This RCT study will provide, for the first time, information regarding the effect of a medication designed to augment cognitive functioning have on the risk of falls in older adults with Mild Cognitive Impairment. We will expect a significant reduction in the risk of falls in this vulnerable population as a function of the reduced gait variability achieved by treatment with cognitive enhancers. In this vein, we would establish that medications that augment cognitive function could be a complementary therapeutic option for reducing fall risk in people with MCI. This may contribute to a new approach to prevent and treat fall risk in this population, which will lead to improving the autonomy and quality of life of seniors in early stage of dementia.

## Competing interests

The authors declare that they have no competing interests.

## Authors' contributions

MMO conceived the study, and drafted the original protocol. JLW, MJB, and MS participated in the design of this study protocol. All authors read and approved the final manuscript.

## Pre-publication history

The pre-publication history for this paper can be accessed here:

http://www.biomedcentral.com/1471-2377/9/42/prepub
